# Potential Role of HMGCS2 in Tumor Angiogenesis in Colorectal Cancer and Its Potential Use as a Diagnostic Marker

**DOI:** 10.1155/2019/8348967

**Published:** 2019-07-01

**Authors:** Kejian Zou, Yan Hu, Musheng Li, Hongli Wang, Yuhua Zhang, Ling Huang, Yuanwen Xie, Songyu Li, Xingui Dai, Wanfu Xu, Zhiyong Ke, Sitang Gong, Yaodong Wang

**Affiliations:** ^1^Department of General Surgery, Hainan General Hospital, Haikou 570311, China; ^2^Department of Anesthesiology, Hainan General Hospital, Haikou 570311, China; ^3^Department of Gastroenterology, Guangzhou Women and Children's Medical Center, Guangzhou Medical University, Guangzhou 510623, China; ^4^Department of Pediatrics, The Ninety-Five Hospital of PLA, Putian 351164, China; ^5^Department of Anorectal, Qionghai Hospital of Traditional Chinese Medicine, Qionghai 571400, China; ^6^Department of Clinical Laboratory, Qionghai Hospital of Traditional Chinese Medicine, Qionghai 571400, China; ^7^Department of Critical Care Medicine, Institute of Transitional Medicine, The First People's Hospital of Chenzhou, Chenzhou 423000, China; ^8^Guangzhou Institute of Pediatrics, Guangzhou Women and Children's Medical Center, Guangzhou Medical University, Guangzhou 510623, China; ^9^Department of Cell Biology, School of Basic Medical, Southern Medical University, Guangzhou 510515, China; ^10^Kunshan Affiliated Hospital of Nanjing University of Chinese Medicine, Kunshan 215300, China

## Abstract

**Objective:**

HMGCS2 is the rate-limiting enzyme of ketogenesis, which is vital for tumor initiation or metastasis. The aim of this study is to determine the relationship between HMGCS2 and tumor angiogenesis.

**Materials and Methods:**

The study consisted of 100 cases with colorectal cancer and healthy control, the expression of HMGCS2 and the microvessel density (MVD) (marker: CD31) were analyzed by immunohistochemistry and tube formation, and the centration of *β*-hydroxybutyrate in serum was assessed by biochemical analysis.

**Results:**

The results showed that HMGCS2 expression is significantly reduced in colorectal cancer compared with healthy control, which is inversely correlated with MVD in colorectal cancer by IHC analysis. What is more, knockdown HMGCS2 expression in HT-29 cells significantly contributed endothelial cell tube formation.

**Conclusion:**

These findings implying HMGCS2 may have a negative regulation of tumor angiogenesis and provide an approach to inhibit tumor angiogenesis.

## 1. Introduction

Colorectal cancer is a characterized as a group of metabolic reprogramming, an emerging hallmark of cancer cells [1], and immune-mediated disorders of intestinal tissue. Enhanced aerobic glycolysis (Warburg effect) is the main metabolic characteristics of malignant tumors [2]. Accumulating evidence showed that metabolic rewiring in cancer not only is limited to glucose metabolism, but also included lipid and ketone body [3, 4]. Emerging evidence indicates that fundamental differences exist between the metabolic pathways of normal and malignant cells [5, 6]. In contrast to normal cells, which derive most of their usable energy through oxidative phosphorylation, cancer cells depend heavily on substrate phosphorylation pathways to meet energy demands [1]. The convincing evidence showed that lactate and succinate, higher than normal levels in tumor cells, promote tumor growth whereas *β*-hydroxybutyrate, lower than normal levels, suppresses tumor growth [7]. Interestingly, the expression of the rate-limiting enzyme in the synthesis of *β*-hydroxybutyrate, 3-hydroxy-3-methyl glutaryl CoA synthase 2 (HMGCS2), is repressed by the oncogene c-Myc [8], and SIRT3, associated with tumor suppression function, is responsible for the maintenance of HMGCS2 in the deacetylated state, thus keeping the enzyme in optimally active form [9]. All these findings point to a tumor-suppressive role for *β*-hydroxybutyrate in colon. However, mitochondria are the sites of multiple metabolism, such as pyruvate oxidation, citric acid cycle, oxidative phosphorylation, ketogenesis, and fatty acid oxidation, which is critical role in tumorigenesis [7]. Dysfunction of mitochondrial function is one hallmark of tumor that directly linked to oncogenesis, angiogenesis, Warburg effect, and epigenetics.

Recently, a large number of evidences showed that HMGCS2, a regulatory point converting acetyl-CoA to ketone bodies in the pathway [10, 11], enhanced invasion and metastasis via direct interaction with PPAR*α* to activate Src signaling in colorectal cancer and oral cancer [12] and represented a potential novel prognostic biomarker for ESCC patients [13]. Expressed in liver and a number of extrahepatic tissues, like colon, HMGCS2 has also been reported to play a crucial preventive role in several cancers, such as rectal cancer, breast cancer, and prostate cancer [14–16]. What is more, a strong correlation between distant metastasis in advanced CRC and tumor metabolism, angiogenesis, and tumor vasculature is one of the primary targets for CRC therapy. Despite the strong research concerning the function of HMGCS2 in tumor, data are lacking in the potential relationship between HMGCS2 and tumor angiogenesis. We aimed to seek evidence to elucidate the possible relationship between HMGCS2 expression and colorectal cancer prognosis. In this study, we aimed to seek evidence to elucidate the possible relationship between HMGCS2 and tumor angiogenesis in development of CRC.

## 2. Materials and Methods

### 2.1. Cell Culture and siRNA Transfection

The HT29 and HUVEC cell line was purchased from the Shanghai Institute of Biological Sciences, Chinese Academy of Sciences (Beijing, China), and cultured in Dulbecco's modified Eagle's medium (Thermo Fisher Scientific, Inc., Waltham, MA) supplemented with 10% fetal bovine serum (Thermo Fisher Scientific, Inc.), The HMGCS2-specific siRNA was purchased from Genepharma (Shanghai, China) used in Hu* et.al* study [17]. The interfering sequences for HMGCS2 were as follows: sense, 5′-GACUUCUACAAACCAAACUtt-3′ and antisense, 5′-AGUUUGGUUUGUAGAAGUCtt-3′. The interfering sequences for nontargeting siRNA were as follows: sense, 5′-UUCUUCGAACGUGUCACGUtt-3′ and antisense, 5′-ACGUGACACGUUCGGAGAAtt-3′, which were used as the negative control. siRNA were transfected with lipofectamine 3000 (Thermo Fisher Scientific, Inc.) according to the manufacturer's instructions.

### 2.2. Patients and Biopsies

Based on the declaration of Helsinki as reflected in a prior approval by the institution's human research committee, this study was conducted in a cohort of patients with colorectal cancer (CRC) in Hainan General Hospital approved by the Medical Ethical Review Board. A number of 100 cases (20 cases healthy control vs 80 cases CRC) were included in this study; the detailed information was supplied in Supplementary Materials Table 1. The intestinal tissue was drawn from each patient by electronic colonoscopy after we got the informed consent from the patients diagnosed with CRC. Written informed consent was obtained from all participants, which are not publicly available since the database is currently not anonymous and contains all patient's name; however, it could be available upon request.

### 2.3. Measurement of *β*-Hydroxybutyrate Level

The blood was collected from the patients with colorectal cancer and analyzed by biochemical analysis to detect *β*-hydroxybutyrate level.

### 2.4. Antibodies and Reagents

PBS and other chemical reagents used in this study were from Sigma (St. Louis, MO, USA), HMGCS2 (E-AB-13296) was from Elabscience (Wuhan, China), and a-tubulin and CD31 (Abclonal, A11525) were purchased from Abclonal (Wuhan, China).

### 2.5. Immunohistochemistry

Slides were heated in an oven for 2 hours at 60°C, dewaxed, distilled with water for 2 minutes, and followed by high pressure steam distillation. After blocking with a peroxidase in 3% H_2_O_2_ solution for 10 minutes, washing 3 times with phosphate-buffered saline (PBS) for 5 minutes, the mixture was supplemented with a primary antibody and incubated at 37°C for 60 minutes. After PBS washing in the same manner, secondary antibodies to HMGCS2 (Elabscience, E-AB-13296) and CD31 (Abclonal, A11525), respectively, were added. After incubation at 37°C for 60 minutes in PBS, samples were washed 3 times with PBS for 5 minutes. Each section was colored by diaminobenzidine for 1 to 2 minutes. Each slice was photographed microscopically, and the images were analyzed by IPP6.0 image analysis software.

### 2.6. IHC Scoring and Microvascular Density

HMGCS2 staining intensities with scores of 0 and 1 were defined as low expression of HMGCS2, while scores of 2 and 3 were defined as high expression. 5–8 fields of view were selected and photographed from each section microvascular density (MVD).

### 2.7. Tube Formation Assay by HUVEC Cocultured with HT29 Cells

The tube formation assay was performed in pervious study [18, 19]. Briefly, tumor cells were cultured on Transwell inserts (12 mm diameter, polycarbonate membranes with 0.4 *μ*m pores; Corning, Lowell, MA, USA). After 24 h the inserts were transferred on top of endothelial cells plated on Matrigel (1.5 × 10^5^ cells in 12-well multiplate). After 8 h of incubation, endothelial cells were photographed and network formation on Matrigel was measured by means of the number of branching points (Nikon Eclipse E400 and camera Nikon DS-5MC).

### 2.8. Western Blotting

Cells were lysed in RIPA buffer (50 mM Tris, 1 mM EDTA, 150 mM NaCl, 1% Triton X-100, 0.1% SDS, and 0.5% sodium deoxycholate) that included protease inhibitors (Roche, Indianapolis, IN). Western blot analysis was performed as described previously [17]. Protein concentrations were measured by using a bicinchoninic acid (BCA) assay kit (Dinguo, Beijing, China). Protein samples were separated by a 10% SDS-PAGE and then transferred to a PVDF membrane (Merck Millipore, Billerica, MA). The membranes were blocked for 1 h in Tris-buffered saline containing 0.05% Tween-20 and 5% nonfat milk and were then probed with the respective primary antibodies against the target protein overnight at 4°C. The blots were then washed and incubated for 1 h at room temperature with horseradish peroxidase-conjugated anti-rabbit or anti-mouse secondary antibodies. Bands were visualized by using an ECL reagent (Thermo, Marina, CA).

### 2.9. RNA Extraction and Real-Time PCR Assay

The total RNA were extracted as described in our previous work [20]. Briefly, total RNA was isolated from tissue using TRIZOL reagent (Invitrogen), and cDNA was synthesized using an All-in-One First-Strand cDNA Synthesis Kit. The relative gene expressions were detected using an All-in-One qPCR Mix. HMGCS2 expression was amplified using commercial primers specific for HMGCS2 (HS00985427-M1) and beta-2-microglobulin (B2M) (HS99999907-M1) was designed by Applied Biosystems (TaqMan® Gene Expression Assays).

### 2.10. Statistical Analysis

All statistical analyses were performed using SPSS 22.0 (SPSS, Inc., Chicago, III). Data were expressed at the mean with standard deviation (SD). All statistical analyses utilized 0.05 levels of significance.

## 3. Results

### 3.1. Patient's Characteristics.

As shown in Table 1, the patients in this study consisted of 80 patients with CRC and 29 healthy controls with regard to tumor grade (consisted of 12 cases stage I (15%), 39 cases stage II (48.75%), 24 cases stage III (30%), and 12 cases stage IV (6.25%)); patients were classified as male and female stage, 56.25% and 43.75%, respectively. In addition, according to the age at point 50 years, 19 cases (46.25%) are less and equal than 50, and 61 cases (53.75%) were greater than 50 years.

### 3.2. *β*-Hydroxybutyrate Level Has No Significant Difference in Patients with Colorectal Cancer and Healthy Control

There is increasing evidence of the therapeutic benefits of artificially induced mild ketosis in various disorders. Some endogenous products of fat metabolism are D-*β*-hydroxybutyrate (BHB), acetoacetate (AcAc), and acetone. To detect the changes of *β*-hydroxybutyrate in patients with colorectal cancer, we collected blood from patients diagnosed with colorectal cancer in clinic and analyzed the concentration of *β*-hydroxybutyrate by biochemical analysis. The results showed no significant change of *β*-hydroxybutyrate level between patients and healthy control.

### 3.3. HMGCS2 Expression Is Significantly Reduced in Patients with Colorectal Cancer

**Figure 1:**
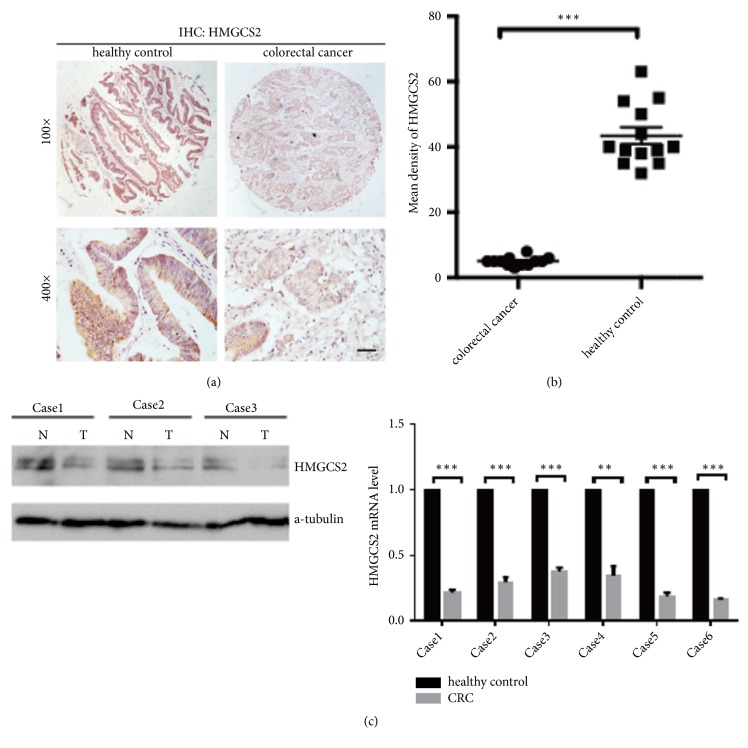
*Attenuation of HMGCS2 expression in colorectal cancer*. (a) IHC was performed to detect HMGCS2 expression in clinical sample from colorectal cancer and healthy control. Scale bar, 50 *μ*m. (b) Statistical analysis of mean density of HMGCS2 expression in indicated group by t test, ∗∗∗*p*<0.001. (c) Western blotting and real-time PCR were used to detect HMGCS2 expression in healthy control and CRC. ∗∗p<0.01 ∗∗∗p<0.001.

Despite above results which showed that no significant difference of *β*-hydroxybutyrate level in patients and healthy control, cellular energy reprogramming is one of the recognized emerging hallmarks of tumor, including tumor glycolysis, lipid, and ketone body metabolism. Ketogenesis is a crucial alternative metabolic pathway that provides lipid-derived energy for various organs during carbohydrate deprivation such as in fasting [21], and ketone bodies may be vital fuel in ketogenesis for tumor initiation or metastasis. We focused our attenuation on HMGCS2 expression, a rate- limiting enzyme that catalyzes the first reaction in ketogenesis, in human colorectal cancer tissue microarray by immunochemistry. As shown in Figures 1(a)-1(b), the results revealed that HMGCS2 expression is significantly reduced; even in undetectable level, in CRC patients comparing with that of healthy controls, further results showed that HMGCS2 expression is reduced in CRC at mRNA and protein level (Figure 1(c)). In addition, according to TCGA data, HMGCS2 expression in colorectal cancer, at mRNA levels, is significantly downregulated in tumors (Supplementary Figures 1A-1B).

### 3.4. HMGCS2 Is Inversely Associated with MVD in Advanced CRC

**Figure 2:**
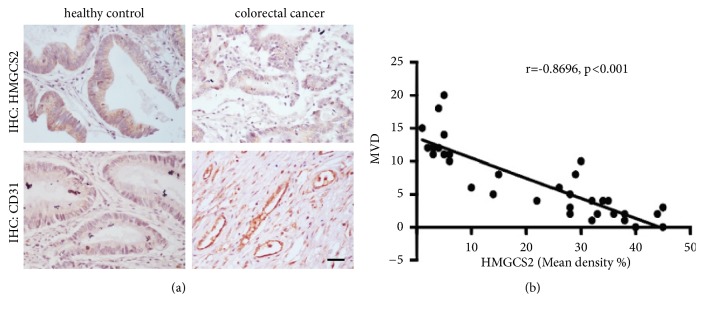
*Negatively correlation of HMGCS2 and angiogenesis in clinical sample.* (a) HMGCS2 and microvessel density (marker: CD31) were showed by IHC assay in colorectal cancer and healthy control tissue. (b) Correlation between HMGCS2 and MVD. Scale bar, 50 *μ*m.

Recent studies have provided evidence that several key proteins in metabolic reprogramming, including PKM2 [22] and FASN [23], play an important in tumor angiogenesis. Another study showed that the expression of proteins associated with angiogenesis and vascular permeability in a mouse glioma model was altered under the ketogenic diet (KD) [24], suggested a potential utility of the KD as an adjuvant treatment for tumors, and highlighted the importance of metabolism in tumor progression. Interestingly, we found enrichment of microvessel density in colorectal cancer tissue with lower HMGCS2 expression (Figure 2(a)). Further analysis showed that HMGCS2 expression was negatively correlated with expression and was correlated with microvessel density (Spearman R=-0.8696, p<0.001) (Figure 2(b)), implying that HMGCS2 is a critical factor involved in tumor angiogenesis.

### 3.5. Inhibition of HMGCS2 Expression in CRC Enhanced HUVEC Tube Formation

**Figure 3:**
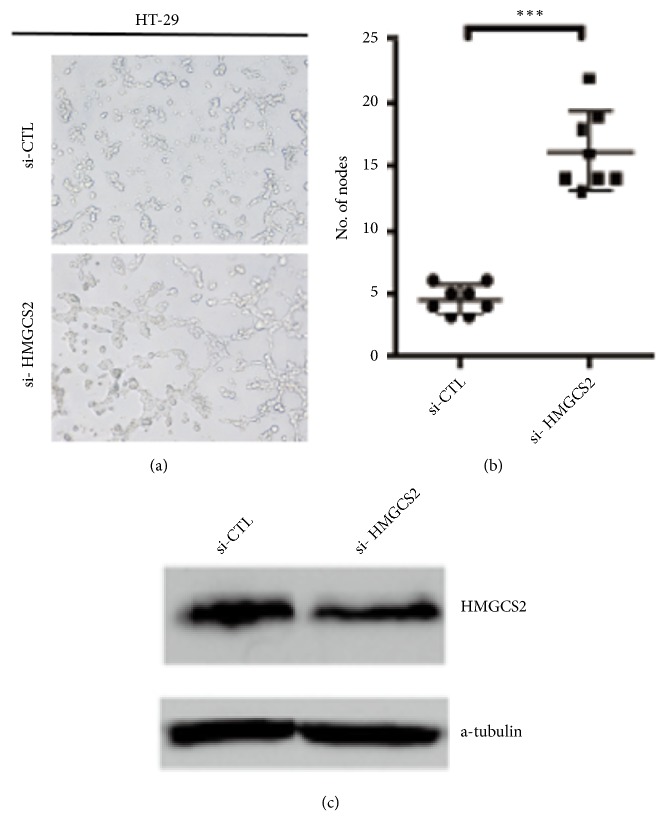
*HMGCS2 inhibited tumor angiogenesis*. (a) HUVECs were treated with conditional medium from HT-29 cells transfected with si-CTL and si-HMGCS2. Tube formation of HUVEC cells was visualized by phase contrast inverted microscope (100×). (b) Tube formation was assessed; the number of nodes per image was quantified and analyzed by two-way ANOVA, ∗∗p < 0.01. (c) Knockdown efficiency of HMGCS2 in HT-29 cells was verified by western blotting.

In order to investigate whether HMGCS2 of colorectal cancer cells regulate HUVEC cells tube formation to strengthen the above results* in vivo*, as shown in Figure 3(a), conditioned media (CM) from HT29 cells transfected with siRNA targeted HMGCS2 significantly increased tube formation compared with CM from HT29 cells transfected with siRNA-NC. The corresponding statistics was shown in Figure 3(b), and knockdown efficiency of HMGCS2 was verified by western blotting. Together, our data indicated that HMGCS2 plays an important role in tumor angiogenesis.

## 4. Discussion

Tumor angiogenesis and tumor metabolic reprogramming, two critical events in development of CRC, have been well documented in a large number of studies. In addition to PKM2 [25, 26], PFKFB3, and FASN [23, 27], there are no available reports about the role of HMGCS2 and angiogenesis in various cancers, including colorectal cancer. In this study, we showed that HMGCS2 expression was significantly reduced in colorectal cancer compared with normal tissue, while no significant difference of *β*-hydroxybutyrate level in blood was obtained between CRC and healthy control. In addition, knockdown of HMGCS2 expression in HT29 cells drastically increased tumor angiogenesis. Reduction of HMGCS2 expression was correlated with tumor angiogenesis in colorectal cancer tissues compared with normal tissue, displaying a negative clinical relationship. These findings indicate that HMGCS2 are crucial mediator of angiogenesis and could be a potential marker.

Cancer cells themselves are also able to reprogram their metabolic pathways in response to shift in cellular energy levels and nutrient status, a process boosted by oncogenic mutations and/or tumor suppressor alterations. Ketone bodies, including 3-hydroxy-butyrate, acetoacetate, and acetone, are naturally occurring mitochondrial fuels that are normally produced in the liver during periods of starvation [28, 29]. Extensive research performed on various tumor cell lines, including gliomas, suggests that they do not utilize ketone bodies as energy substrates, but rather as precursors for lipid synthesis, and further that some lack the enzymatic machinery for ketone body metabolism [30–34]. Other reports demonstrated that cancer cells do express at least some ketolytic enzymes and retain the ability to metabolize ketone bodies [35, 36]. In this study, we reported that HMGCS2 expression was lower in colorectal cancer* compared with normal tissue* by IHC, western blotting and real-time PCR, which is consisted with the results in Zhang* et al. [37]* and Camarero N* et al. [8] *lab. In addition, a study by Chen et al. showed that HMGCS2 expression was increased* in advanced TNM stage* based on tumor stage by real-time PCR analysis [12], implying that HMGCS2 expression is associated with differentiation, which has been reported by Wang et al. study [38]. What is more, according to TCGA data analysis, HMGCS2 expression in colorectal cancer, at mRNA levels, is significantly downregulated in tumors based on sample types (Supplementary Figure 1A), individual cancer stages (Supplementary Figure 1B), and histological subtype (Supplementary Figure 1C).

In addition, data from TCGA showed that CRC patients with low HMGCS2 expression survived significantly shorter than those with high HMGCS2 expression (Supplementary Figure 1D); in line with this, our results exhibited the similar results by kaplan–Meier survival curves (Supplementary Figure 1E). These findings indicated that HMGCS2, which served as a tumor suppressor gene, not only was downregulated in colorectal cancer compared with healthy control, but also was associated with tumor differentiation. However, there is no significant difference of *β*-hydroxybutyrate level in blood in patients with colorectal cancer or healthy control; this phenomenon may partly attribute to whether in empty state and tissue ketolysis, the opposite process to ketogenesis in very specialized transformed cells, carried out in almost all cells. The work was further required to elucidate this issue.

In order to continue to grow and disseminate, tumor must acquire a new blood supply. Neovascularization can be enacted by a number of different mechanisms. Our work revealed that HMGCS2 expression was drastically reduced in colorectal cancer tissue with enriched microvascular stained by CD31, and* in vitro* results found that depletion of HMGCS2 expression by siRNA transfection significantly increased tube formation. However, the further work is required to elucidate the mechanism underlying the influence of HMGCS2 on angiogenesis.

## 5. Conclusion

This is the first study to delineate the molecular and clinical characteristics of HMGCS2 in CRC. HMGCS2 expression was strongly reduced in CRC and negatively correlation with MVD, predicting a poor prognosis of CRC patients.

## Figures and Tables

**Table 1:** The characteristic of patients with colorectal cancer.

Variables	Number of patients (%)	*β*-hydroxybutyrate (mean ± SEM)∗

Total	80	
Age		
<=50 years	19 (46.25%)	0.2458 ± 0.0612
>50 years	61 (53.75%)	0.3475 ± 0.0615
Gender		
Male	45 (56.25%)	0.2929 ± 0.0477
Female	35 (43.75%)	0.3471 ± 0.0794
Stage		
I	12 (15%)	0.0900±0.0427
II	39 (48.75%)	0.1700±0.0802
III	24 (30%)	0.1900±0.0726
IV	5 (6.25%)	0.330±0.2874

∗Normal level is 0.03-0.3.

## Data Availability

The data used to support the findings of this study are available from the corresponding author upon request.

## Abbreviations

AcAc: Acetoacetate
BHB: D-*β*-Hydroxybutyrate
CoAD: Colon adenocarcinoma
CRC: Colorectal cancer
ESCC: Esophageal squamous cell carcinoma
FASN: Fatty acid synthase
HMGCS2: 3-Hydroxy-3-methylglutaryl-CoA synthase 2
IHC: Immunohistochemistry
MVD: Microvessel density
PKM2: Pyruvate kinase isozymes M2
PPAR*α*: Peroxisome proliferator-activated receptors *α*
PFKFB3: 6-Phosphofructo-2-kinase/fructose-2,6-bisphosphatase 3
TCGA: The Cancer Genome Atlas.

## Supplementary Materials

Supplementary Materials Supplementary Figure 1: HMGCS2 expression and associated with survival in colorectal cancer. According to TCGA data, HMGCS2 expression, at mRNA levels, was reduced in colorectal cancer based on sample types (A), individual cancer stages (B), and histological subtypes (C). Survival curve was performed to analyze based on HMGCS2 expression in colorectal cancer from TCGA data (D) and IHC results (E). Supplementary Table 1: the characteristic of patients with CRC.
