# Supplementary figures and images for: Potential Role of HMGCS2 in Tumor Angiogenesis in Colorectal Cancer and Its Potential Use as a Diagnostic Marker
Source: Can J Gastroenterol Hepatol. 2019 Jul 1;2019:8348967. doi: 10.1155/2019/8348967 (PMC6634068; doi:10.1155/2019/8348967)

**Supplementary Fig.1**


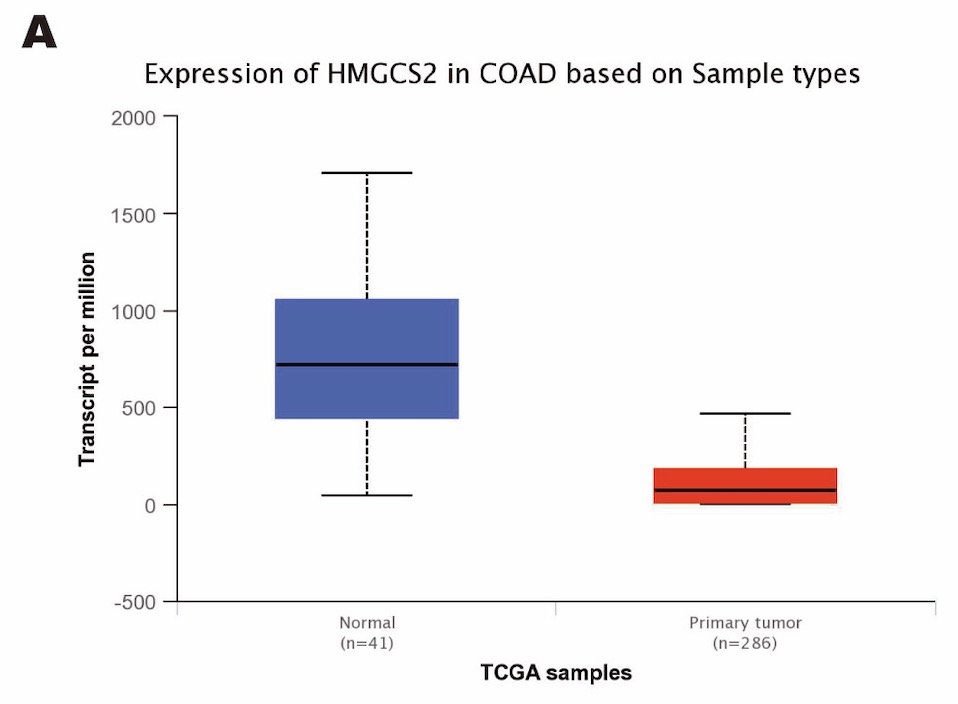

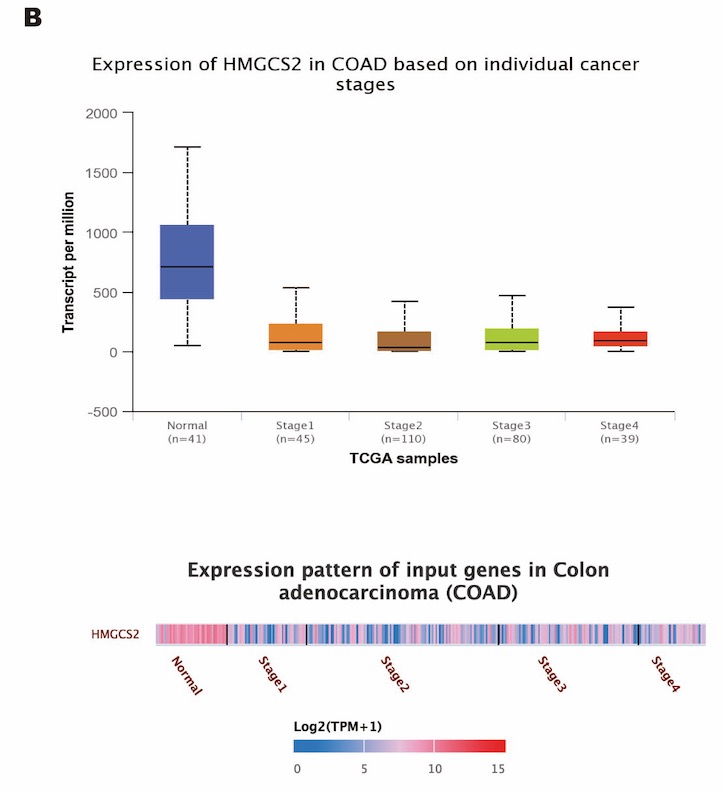

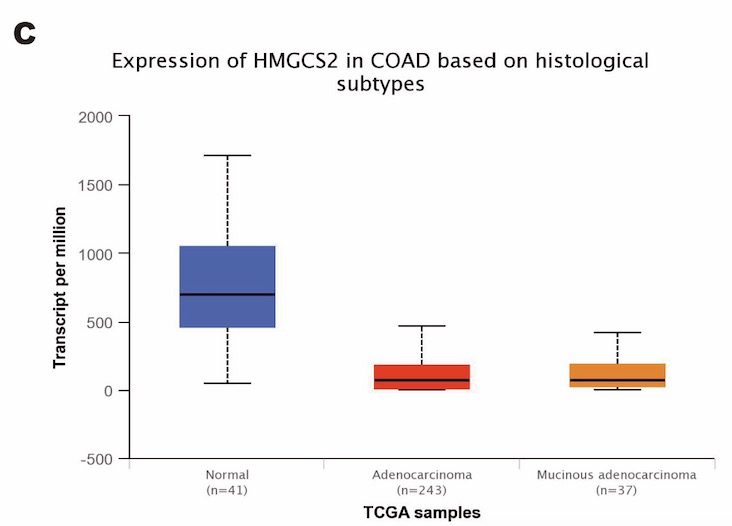

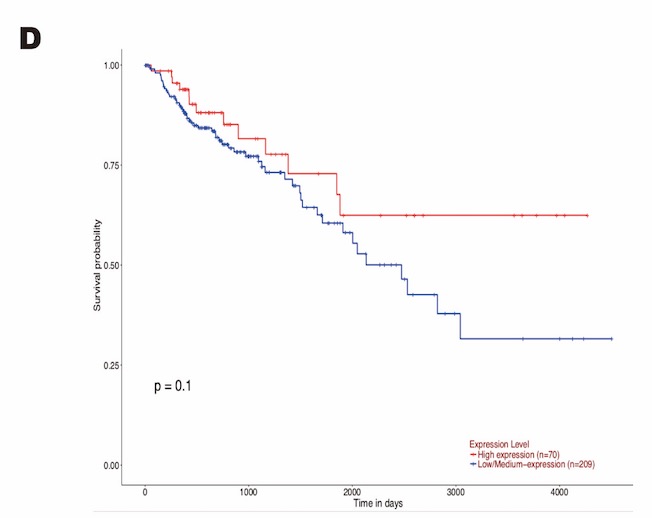

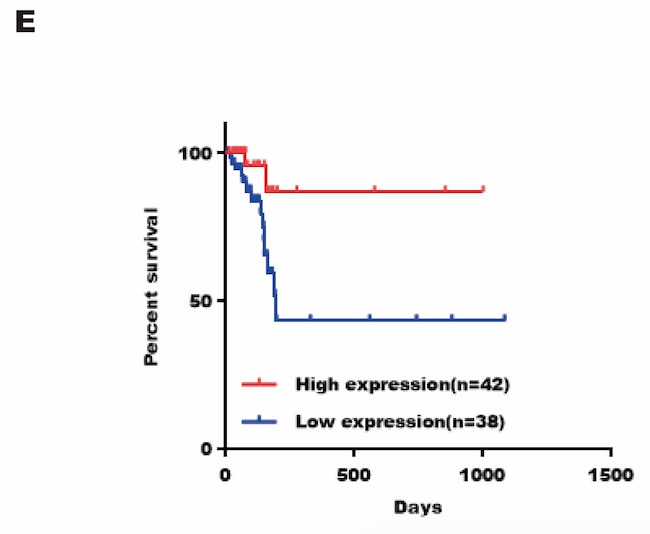

Supplement: Supplementary Materials — Supplementary Figure 1: HMGCS2 expression and associated with survival in colorectal cancer. According to TCGA data, HMGCS2 expression, at mRNA levels, was reduced in colorectal cancer based on sample types (A), individual cancer stages (B), and histological subtypes (C). Survival curve was performed to analyze based on HMGCS2 expression in colorectal cancer from TCGA data (D) and IHC results (E). Supplementary Table 1: the characteristic of patients with CRC. [file 8348967.f1.zip › 8348967.f1.docx]
